# Muscle volume imbalance may be associated with static posterior humeral head subluxation

**DOI:** 10.1186/s12891-021-04146-3

**Published:** 2021-03-15

**Authors:** Marian Mitterer, Nicholas Matis, Gernot Steiner, Imre Vasvary, Reinhold Ortmaier

**Affiliations:** 1 Department of Orthopaedics and Traumatology, Paracelsus Medical University Salzburg, 5020 Salzburg, Austria; 2 Department for Radiology, Private Hospital Wehrle-Diakonissen, 5020 Salzburg, Austria; 3 Department of Orthopedic Surgery, Ordensklinikum Barmherzige Schwestern Linz, Vinzenzgruppe, Center of Orthopedic Excellence, Teaching Hospital of the Paracelsus Medical University Salzburg, Seilerstätte 4, 4010 Linz, Austria; 4 Institute for Sports Medicine, Alpine Medicine and Health Tourism (ISAG), Tirol Kliniken GmbH, Innsbruck and UMIT, 6060 Hall, Austria

**Keywords:** Rotator cuff, Subluxation, Arthritis, Muscle imbalance, Shoulder

## Abstract

**Background:**

The transverse force couple (TFC) of the rotator cuff (subscapularis vs. infraspinatus and teres minor muscle) is an important dynamic stabilizer of the shoulder joint in the anterior-posterior direction. In patients with posterior static subluxation of the humeral head (PSSH), decentration of the humeral head posteriorly occurs, which is associated with premature arthritis. We hypothesize that not only pathologic glenoid retroversion but also chronic muscle volume imbalance in the transverse force couple leads to PSSH.

**Methods:**

A retrospective analysis of the TFC muscle volumes of 9 patients with symptomatic, atraumatic PSSH, within 8 were treated with glenoid correction osteotomy, was conducted. The imaging data (CT) of 9 patients/10 shoulders of the full scapula and shoulder were analyzed, and the muscle volumes of the subscapularis (SSC), infraspinatus (ISP) and teres minor muscles (TMM) were measured by manually marking the muscle contours on transverse slices and calculating the volume from software. Furthermore, the glenoid retroversion and glenohumeral distance were measured.

**Results:**

The mean glenoid retroversion was − 16° (− 7° to − 31°). The observed mean glenohumeral distance was 4.0 mm (0 to 6.8 mm). Our study population showed a significant muscle volume imbalance between the subscapularis muscle and the infraspinatus and teres minor muscles (192 vs. 170 ml; *p* = 0.005). There was no significant correlation between the subscapularis muscle volume and the glenohumeral distance (*r* = 0.068), (*p* = 0.872).

**Conclusion:**

The muscle volume of the SSC in patients with PSSH was significantly higher than the muscle volume of the posterior force couple (ISP and TMM). This novel finding, albeit in a small series of patients, may support the theory that transverse force couple imbalance is associated with PSSH.

**Level of evidence:**

Level 4 – Case series with no comparison group.

## Background

PSSH leads to eccentric loading on the glenoid and arthritis [1]. Walch described and classified the different morphologies of glenohumeral arthritis in contrast with PSSH in the axial plane [2]. Typically, the classes comprised B1, which corresponds to PSSH together with posterior joint space narrowing, and B2, which corresponds to PSSH and retroverted glenoid with posterior biconcave glenoid formation. Recently, the classification has been extended to include B0 [3], which corresponds to a prearthritic state of PSSH without signs of arthritis on the glenoid side. It appears logical that classes B0 to B3 correspond to chronologic progression over time, with B0 indicating a prearthritic condition that progresses to B1 and finally to B3. However, there is no clear scientific evidence supporting this hypothesis.

There have been several surveys analyzing the reasons for PSSH lately, but it is still not clear what definitely causes PSSH. However, a combination of bony- and soft-tissue factors are thought to be associated with PSSH [1, 3–5]. There is some evidence that increased glenoid retroversion causes PSSH, but it has also been reported that there is no correlation between PSSH and glenoid retroversion. Furthermore, patients who have undergone shoulder surgeries via the open anterior approach are more likely develop PSSH due to tightness of the anterior capsule, which causes the humeral head to migrate posteriorly [6–10]. Furthermore, the rotator cuff, a dynamic stabilizer of the shoulder, plays an important role in stabilizing the shoulder. In an intact, healthy shoulder, the TFC is balanced [11], meaning that the force vectors of the SSC, and the external rotators (ISP muscle and TMM), are directed through the center of the glenoid. If there is a mismatch, the resulting force vector changes. Therefore, we hypothesize that in patients with PSSH, the subscapularis muscle is disproportionate in volume and in strength compared to the infraspinatus and teres minor muscle. To verify this hypothesis, we compared the muscle volumes of the subscapularis, infraspinatus and teres minor, as well as glenohumeral distance in the shoulder, in patients with PSSH and pathological glenoid retroversion.

## Methods

We compared the muscle volumes of the subscapularis muscle and the combined volume of the infraspinatus and teres minor muscle. Furthermore, the correlation between a muscular imbalance and concomitant retroversion of the glenoid as well as glenohumeral distance was assessed.

Therefore, we retrospectively reviewed the charts of patients who were surgically and conservatively treated for symptomatic, atraumatic PSSH and advanced glenoid retroversion.

In the study period from 2008 to 2016, we included 10 shoulders in 9 patients with PSSH, within 8 patients were treated with glenoid correction osteotomy. The PSSH cases were classified by CT images. The mean age of the patients was 43.5 years (range, 22 to 56). All of the patients were men.

Eight of these patients have previously been included in a study about correction osteotomy for treating excessive retroversion of the glenoid and PSSH in younger patients [12]. The remaining patient was treated conservatively (Table 1).

**Table 1:** Demographic data of the study population

	Sex	Age	Surgery	Affected side
Shoulder. 1	M	36	Yes	Right
Shoulder. 2	M	28	Yes	Right
Shoulder. 3	M	54	Yes	Right
Shoulder. 4	M	54	Yes	Left
Shoulder. 5	M	56	Yes	Right
Shoulder. 6	M	45	Yes	Left
Shoulder. 7	M	46	Yes	Left
Shoulder. 8	M	53	Yes	Right
Shoulder. 9	M	38	Yes	Right
Shoulder. 10	M	22	No	Left

In all 9 patients (10 shoulders), CT scans were used for analysis. For the 8 patients who were treated surgically, only preoperative CT scans were used for evaluation. In all patients, a full scapula CT scan was available.

**Fig. 1:**
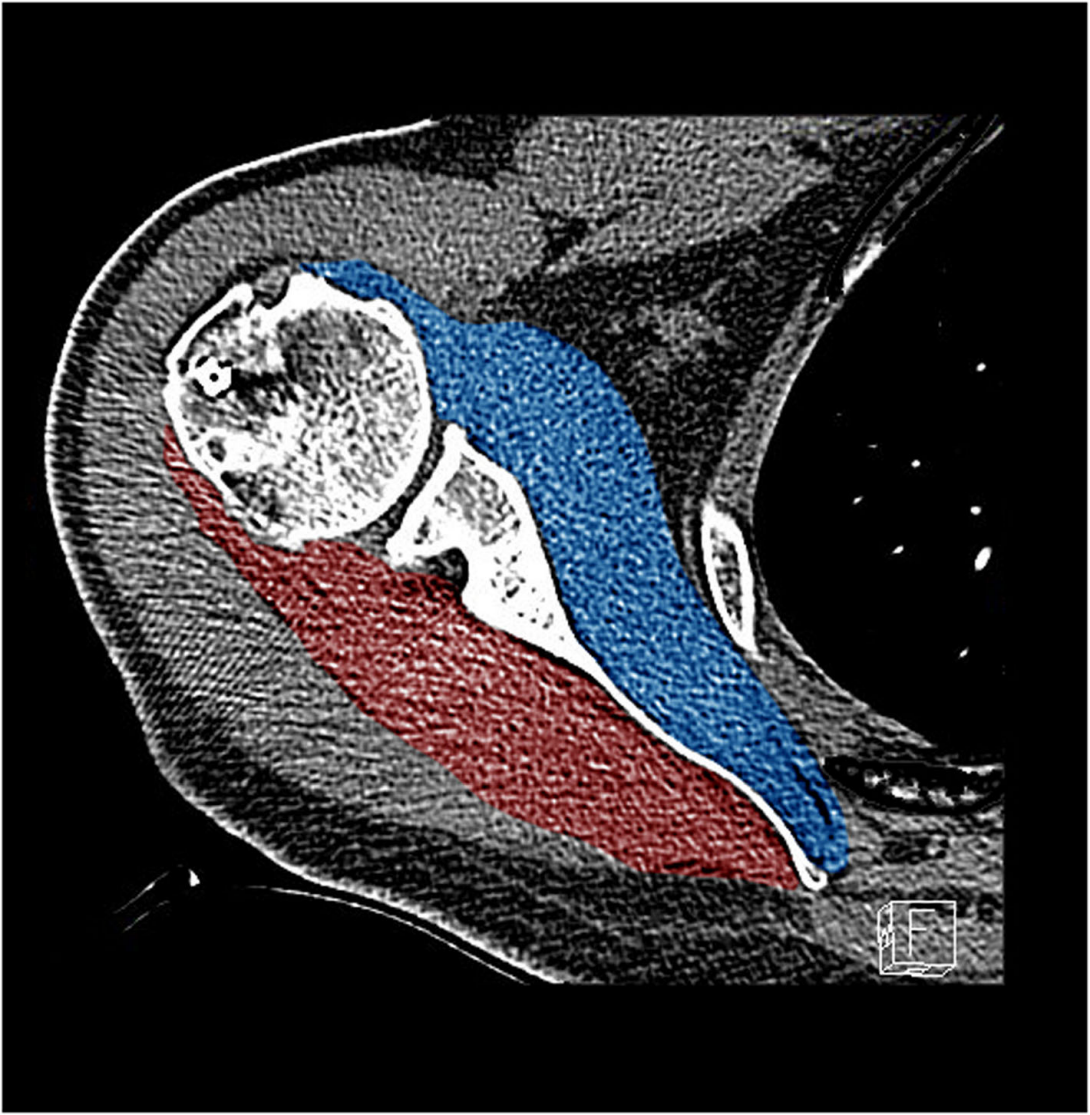
Calculations of the SSC (blue) and ISP/TM (red) volumes (193 ml vs. 185 ml) of the right shoulder of a 56-year-old male

To measure the muscle volume of the transverse force couple, we used the technique described by Piepers et al. [13] Therefore, the muscle contours of the SSC and the ITM were manually marked on every transverse slice, and the muscle volume was calculated automatically from the software (Fig. 1). The reliability of this measurement method has been described by Tingart MJ et al. [14]

All measurements for each patient were performed by two individual observers, and the interobserver correlation coefficient was calculated by comparing the measurements of a muscle contour on a transverse slice.

Glenoid retroversion was calculated using the technique described by Moroder et al. [15], and humeral subluxation (glenohumeral distance) was measured by the technique described by Ortmaier et al. [12]

Furthermore, the muscle volume ratio was calculated as described previous by Espinosa-Uribe et al [11]

### CT scans and measurement technique

CT scans were performed on one of two multislice (MS) CT scanners at our institution: the 128-MSCT Siemens AS+ (Siemens Healthcare, Erlangen, Germany) or 64-MSCT Philips Brilliance (Philips Healthcare, Best, The Netherlands). The scanner used for each of the patients was determined randomly.

The patients were examined in the supine position with their arms adducted and lying on the belly, and the patients were stabilized with a strap around the body.

The CT scans were performed with the standard CT protocol for the shoulders used at the institution, with a slice thickness of 1 mm and 0.7 mm increments with reformations in the axial and paracoronal planes of 2/2 mm.

Muscle volumes were calculated using the postprocessing software program Philips IntelliSpace Portal (Radiology DICOM image processing application software Version 8.0 LOT 8.0.3.30150, 2016-10-17, Philips Medical Systems Nederland B.V).

### Statistical analysis

Statistical analysis was performed using SPSS for Windows, version 25. Associations of muscle volume and glenoid retroversion were calculated using the Pearson correlation coefficient and the paired sample t-test. Differences were considered statistically significant if *p* < 0.05.

## Results

In our patient cohort, the mean muscle volume for the infraspinatus muscle and teres minor muscle was 170 ml (range, 147 to 239 ml). The corresponding muscle volume for the subscapularis muscle was 192 ml (range, 138 to 254 ml) and in comparison, significant higher (170 vs. 192 ml; *p* = 0.005). The observed muscle volume ratio was 1.14 ± 0.13.

The glenoid retroversion ranged from − 7° to − 31° (mean − 16°) and the mean glenohumeral distance was 4.0 mm (range, 0 to 6.8 mm) (Table 2).

**Table 2:** Muscle volume, glenoid retroversion and glenohumeral distance of all participating patients. Shoulders 3 and 4 belong to patient Nr. 3. All calculations were performed on preoperative CT scans

	SSC Muscle-Volume [ml]	ISP/TM Muscle- Volume [ml]	Glenoid retroversion	Glenoid morphology	Glenohumeral distance [mm]
Shoulder 1	228	196	−23°	B1	5
Shoulder 2	138	135	−22°	B2	0
Shoulder 3	165	136	- 14°	B2	4
Shoulder 4	185	147	- 17°	B1	4
Shoulder 5	180	147	- 13°	B1	7
Shoulder 6	190	186	- 14°	B2	7
Shoulder 7	254	239	- 7°	B1	1
Shoulder 8	193	185	- 9°	B1	1
Shoulder 9	205	149	- 31°	B3	6
Shoulder 10	180	175	- 14°	B2	5

**Fig. 2:**
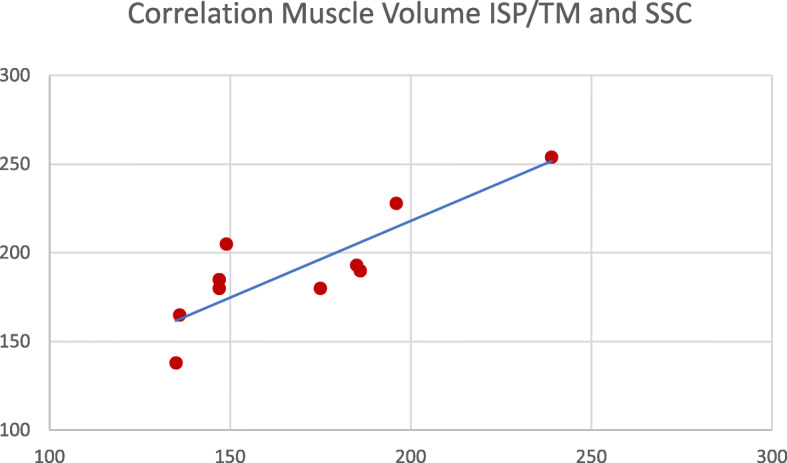
The ISP/TM and SSC volume show a strong positive relationship: Pearson *r* = 0.872

There was a strong positive correlation (*r* = 0.872), (*p* = 0.005) between the volume of the subscapularis muscle and the volume of infraspinatus and teres minor muscles (Fig. 2) indicating an increasing muscle volume imbalance the larger the volume of the SSC is.

The interobserver variability for infraspinatus and teres minor muscle showed a positive correlation (*r* = 0.60), (*p* = 0.049). For the subscapularis muscle the corresponding pearson correlation showed a large positive correlation (*r* = 0.95), (*p* = 0.001).

## Discussion

As hypothesized, patients with PSSH not only show large glenoid retroversion but also a significant muscle imbalance regarding the transverse force couple. In our patient cohort, the muscle volume of the subscapularis muscle was significantly larger than that of the posterior muscle of the infraspinatus and teres minor in every patient.

A larger anterior muscle volume may result in an increased posterior force vector during contraction pushing the humeral head repetitively posterior and contribute to the permanent posterior displacement of the humeral head and therefore to PSSH. Physiologically, it seems that the transverse force couple is balanced, meaning that the muscle volume (which correlates to the force) of the subscapularis muscle and infraspinatus plus teres minor muscle are not significantly different in healthy, nonpathologic shoulders [13].

More specifically, Espinosa-Uribe AG et al. [11] conducted a previous study on 304 shoulders and observed a constant rotator cuff transverse force couple volume ratio of 1.02 ± 0.18 without significant differences across all age and sex groups. Similar results in nonpathologic shoulders have been described by Piepers et al., showing no significant differences between the muscle volume of the anterior (subscapularis) and posterior parts (teres minor/infraspinatus) of the TFC [13].

In our study population, the corresponding volume ratio (SSC vs. ISP/TMM) was 1.14 ± 0.13.

We therefore suppose that a comparatively larger subscapularis muscle creates a posterior directional force when tensioned and pushes the humeral head posteriorly. We think that the posterior force produced by the subscapularis muscle is due to the anatomic way the subscapularis muscle is situated at the back of the thoracic wall with the weakest direction backwards. A similar type of posterior humeral head displacement has been observed in patients undergoing open anterior approach shoulder surgery with postoperative anterior capsular cicatrization. In this study, the muscle volume of the infraspinatus and teres minor muscles negatively correlated with the glenohumeral distance, which may be interpreted as the smaller the muscle volume the higher the amount of (posterior) humeral head displacement. This result supports our hypothesis, that a higher muscle volume imbalance in favor of the SSC worsens PSSH. However, no correlation between the subscapularis muscle volume and glenohumeral distance was found.

Furthermore, we found a negative correlation of glenoid retroversion with the muscle volume of the infraspinatus and teres minor muscles. Retroversion and the muscle volume of the TFC seem influence each other; however, it is not clear whether more retroversion leads to a smaller muscle volume or a smaller muscle volume leads to more retroversion.

The results of this study raise interesting questions. For example, does strengthening of the external rotators positively influence humeral head recentration? Does paralyzing the subscapularis (e.g., with Botox) positively influence humeral head recentration? In our study about correction osteotomy of the glenoid in patients with PSSH [12], we found that retroversion was corrected very well; however, PSSH was not reversed significantly in most of the patients. Taking into account the results of this study, the TFC might be an important future target in treating PSSH. However, there is no evidence for one of the above-mentioned treatment suggestions, and no conclusions can be drawn from the discussed questions. Of course, this study has several limitations, such as the small cohort, as well as unequal distributions of the sexes. Another limitation of this study is the absence of a control group, which can be included in future studies to assess the muscle volume of the transverse force couple in nonpathologic shoulders. However, analyses of the rotator cuff muscle volume in healthy study populations has already been reviewed in several studies, showing no significant difference between the muscle volume of the anterior (subscapularis) and posterior parts (teres minor/infraspinatus) of the transverse force couple [11, 13].

Furthermore, PSSH is very rare, and often, no full scapula CT or MRI scans are available for accurate measurements of the whole muscle volume of the TFC.

Nevertheless, a study with a larger, more homologous cohort and a corresponding control group should be conducted to strengthen the results and prove our theory that muscle volume imbalances may have a positive association with PSSH.

## Conclusion

According to our study, patients with PSSH not only show distinct glenoid retroversion but also a comparatively high subscapularis muscle volume. This novel finding, albeit in a small series of patients, may support the theory that transverse force couple imbalance is associated with PSSH. We therefore suggest that MRI or CT scans are performed to verify not only glenoid retroversion but also the muscle volume corresponding to the transverse force couple in patients with PSSH before initiating therapy or operative treatment.

## Data Availability

The datasets used and/or analyzed during the current study are available from the corresponding author on reasonable request.
